# Case Report: Multiple hemorrhagic metastases to the brain from primary lung choriocarcinoma

**DOI:** 10.12688/f1000research.11681.1

**Published:** 2017-05-23

**Authors:** Sunil Munakomi

**Affiliations:** 1 Department of Neurosurgery, Nobel Teaching Hospital, Biratnagar, Nepal

**Keywords:** primary, lung, choriocarcinoma, brain, metastasis

## Abstract

Herein we report a very rare entity of multiple hemorrhagic metastases to the brain from a primary lung choriocarcinoma in a young woman. The patient presented with recent onset of progressive headache, decreased level of consciousness and multiple episodes of vomiting. CT of the head revealed multiple hemorrhagic lesions within the brain. The patient’s serum B-human chorionic gonadotrophin was increased.  A chest X-ray revealed a right lung mass. The patient urgently underwent operative excision of the lesion in the posterior fossa, so as to prevent impending tonsillar herniation. The histology from the lesion provided the diagnosis of choriocarcinoma. After surgery, ultrasonography of the abdomen and pelvis was normal, and a chest CT revealed an enhanced and highly vascular right apical lung lesion, suggestive of lung primary choriocarcinoma, with regard to the clinical background. The patient was then started on chemotherapy, following which her serum B-HCG level decreased rapidly. This case highlights the importance of keeping this entity in the differential diagnosis of hemorrhagic lesions in any patients of a child bearing age. Early diagnosis and rapid initiation of multimodal therapy is prudent for ensuring a good outcome from an otherwise rapidly metastasizing and highly vascular lesion.

## Introduction

Primary lung choriocarcinoma is an extremely rare entity ^1^. Choriocarcinoma is the malignant proliferation of the syncytial cells of trophoblastic origin following gestational events, such as a term pregnancy, molar pregnancy or an abortion. We herein report one such rare case of multiple hemorrhagic metastases to the brain from primary lung choriocarcinoma in a 22 year old young woman. We also review the literature regarding primary lung choriocarcinoma and discuss recent advancements in the management of this disease.

## Case report

**Figure 1.**
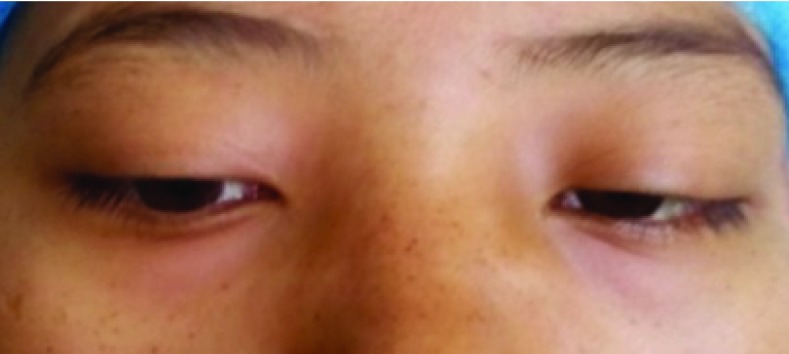
Clinical image showing bilateral sixth nerve palsy in the patient.

A 22 year old woman presented to our emergency department with a history of a recent onset progressive headache for 15 days, followed by decreased level of consciousness and multiple episodes of vomiting for the last 5 days. The patient had a history of normal vaginal delivery one month past. The patient had no history of fever, chills or any rigor associated with these symptoms, and there was no history of abnormal discharge or bleeding from the vagina. There was no other significant past medical and surgical illnesses or any relevant family history. On presentation, the patient was slightly drowsy with a Glasgow Coma Scale of E4V1M3, with bilateral pupils equal and reacting. She had bilateral sixth nerve palsies (left >> right; Figure 1). Neck rigidity was absent. There was no pallor or any lymphadenopathy. Remaining systemic examination was normal. Pelvic and genital examination from a gynecologist did not reveal any abnormal findings.

**Figure 2.**
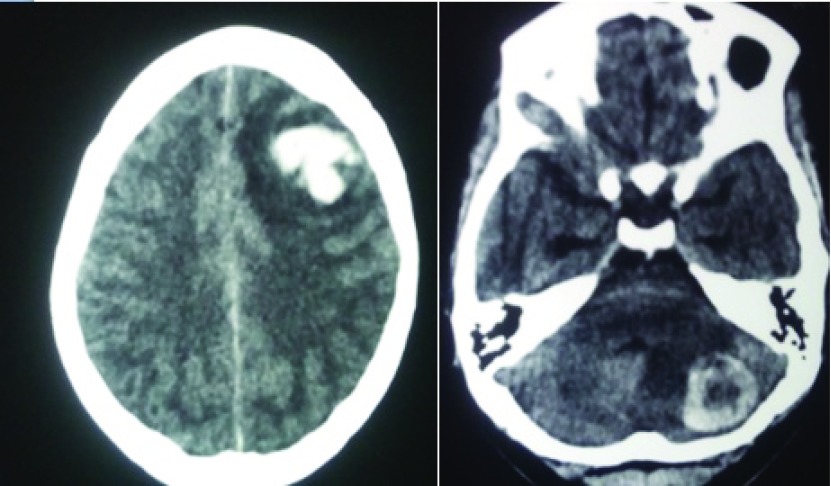
CT head images showing multiple hemorrhagic lesions within the brain.

**Figure 3.**
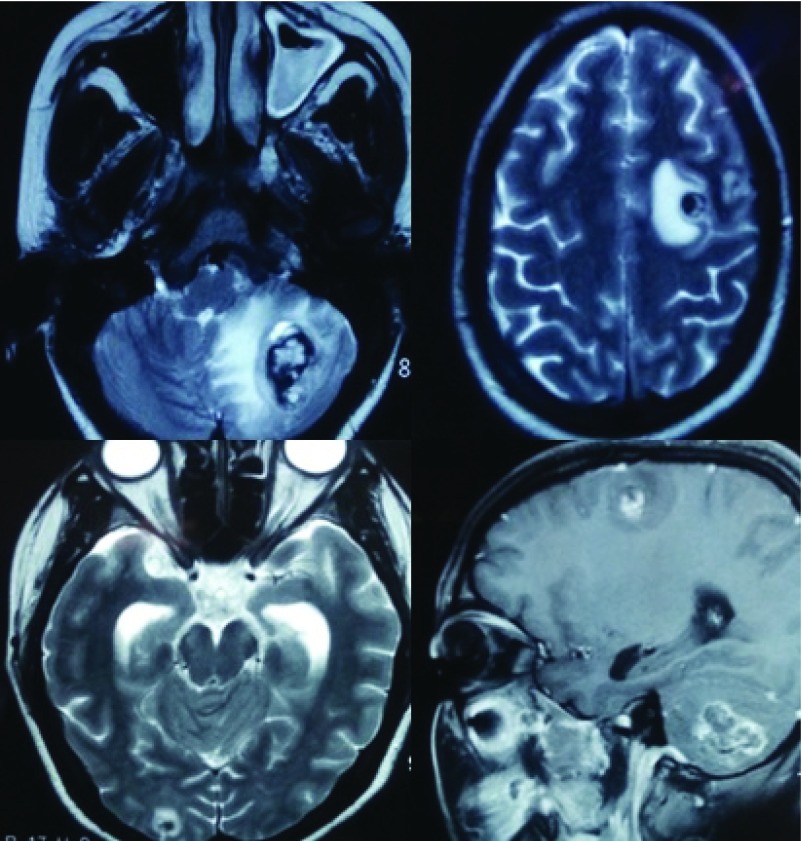
MRI images showing multiple lesions within the brain at different phase of resolution.

**Figure 4.**
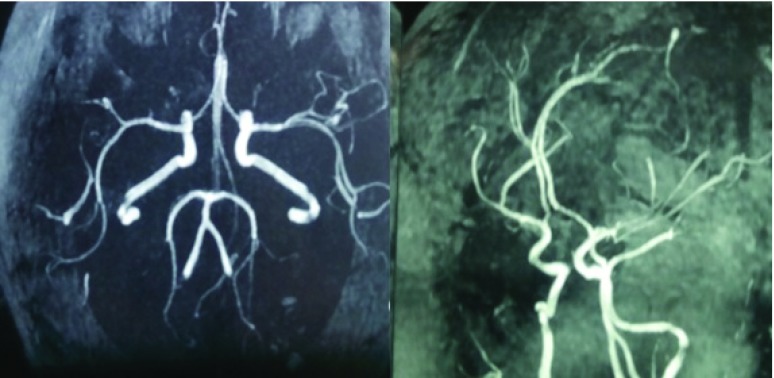
MRI angiography showing absence of any vascular blush or major arterial feeder to the lesions.

**Figure 5.**
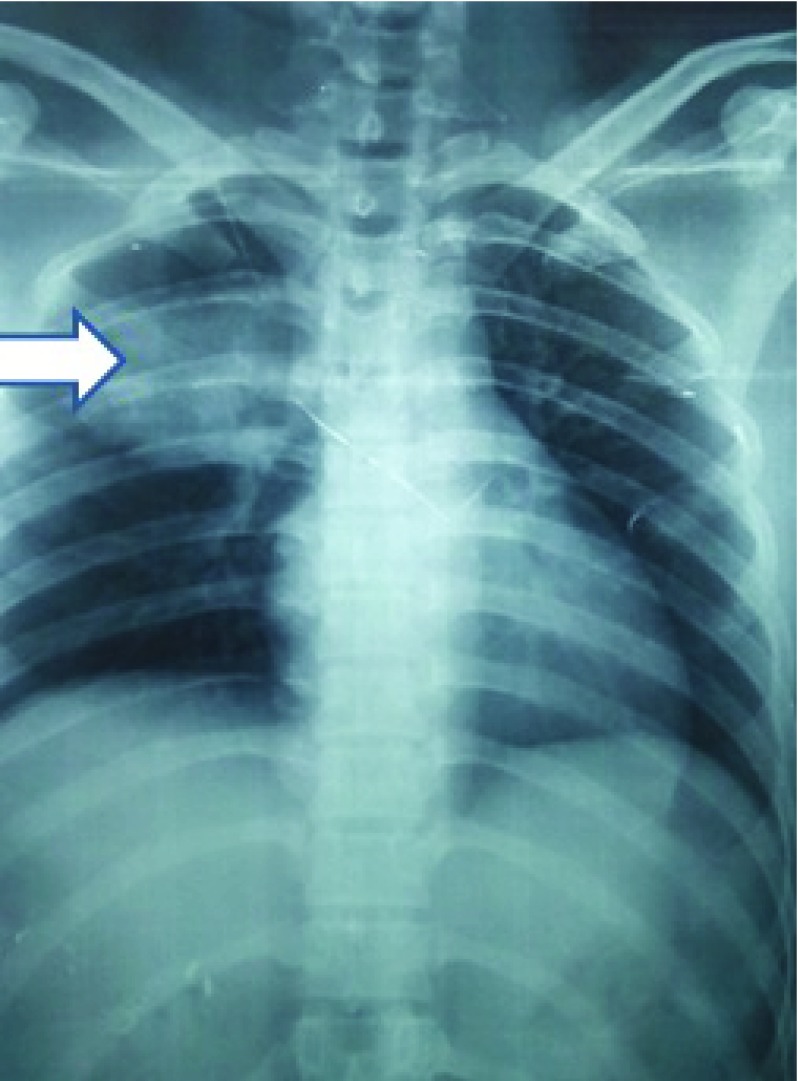
Chest X-ray revealing right sided apical lung lesion.

CT and MRI images of the head revealed multiple hemorrhagic lesions both in the supra and the infra-tentorial compartment with evidence of effacement of the forth ventricle and evolving hydrocephalus ( Figure 2 and Figure 3). There was no vascular blush seen within the brain in the MR angiography ( Figure 4). Routine chest X-ray revealed the presence of a right lung mass ( Figure 5). Urine for pregnancy test was also positive. However, an ultrasound of the abdomen and pelvis was normal. Therefore, choriocarcinoma was suspected and serum B-human chorionic gonadotropin (HCG) levels were assessed, >2,20,000 mIU/ml (normal range: <1 mIU/ml). The patient’s hemoglobin was 14.5 gm% (normal range: 12.1–15.1 gm%) and a platelet count of 2,15,000 (normal range: 1,50,000–4,00,000). Peripheral smear for cytology was normal. Her immune status was normal.

Consequently, a differential diagnosis of multiple hemorrhagic metastases to the brain from the primary lung choriocarcinoma was made. The patient’s husband was informed about the disease condition and the immediate need for the removal of the posterior fossa lesion in order to prevent tonsillar herniation. The patient was in a poor medical condition, so could not decide on her treatment plan.

**Figure 6.**
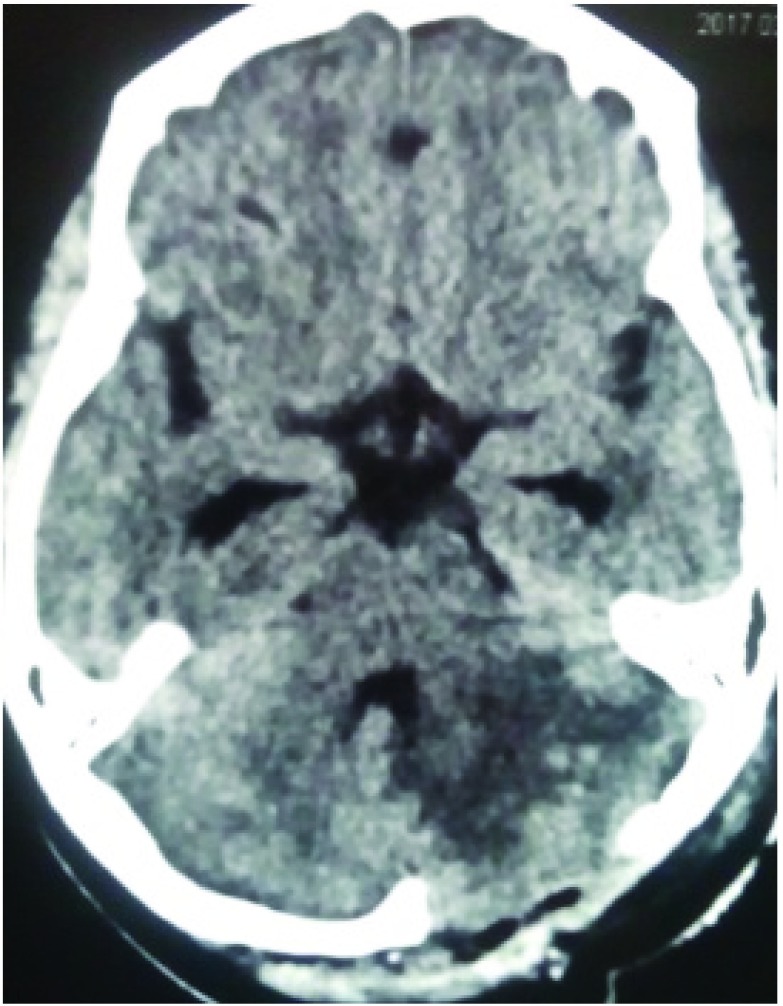
Post-operative CT image showing complete excision of the lesion in the posterior fossa.

The patient immediately underwent sub-occipital craniactomy and excision of the well capsulated hemorrhagic lesion from the left cerebellar hemisphere ( Figure 6). The patient made an uneventful recovery from the surgery and wound sutures were removed on the seventh day.

**Figure 7.**
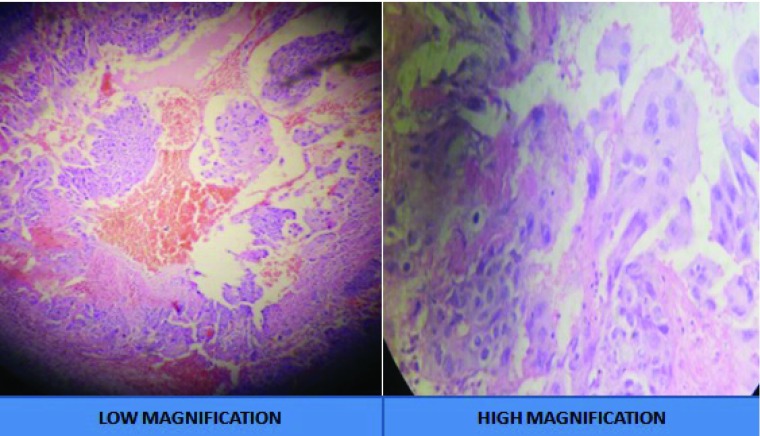
Histology image confirming the diagnosis of invasive choriocarcinoma.

Histopathological study of the excised lesion showed diffuse cohesive sheets of trimorphic malignant trophoblasts, consisting of intermediate trophoblasts and cytotrophoblast, and rimmed with syncytiotrophoblast with the presence of a central hemorrhage and necrosis ( Figure 7). The cells showed striking cytological atypia, high mitotic activity and absence of villi consistent to choriocarcinoma.

**Figure 8.**
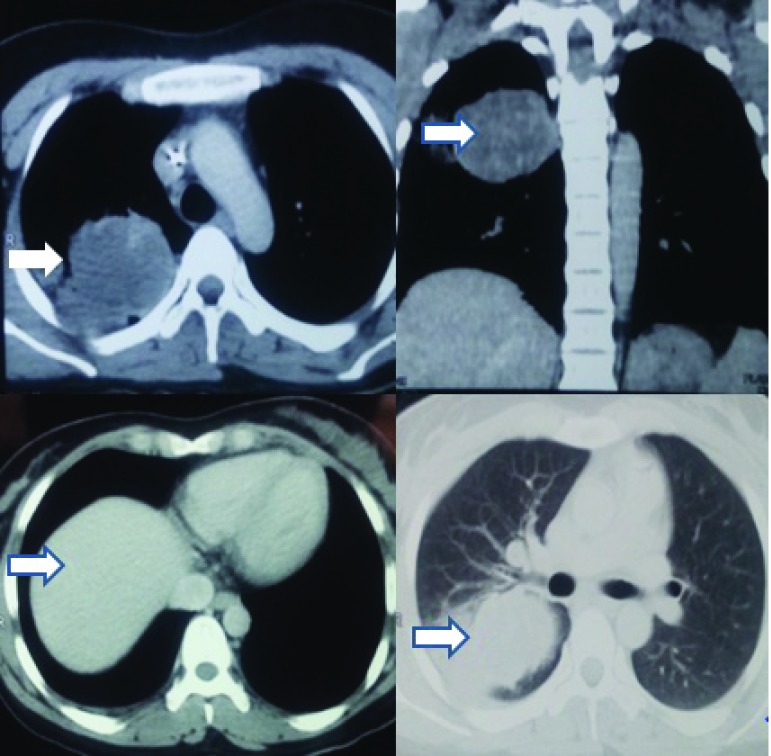
CT chest confirming presence of an enhanced and highly vascular lesion in the upper lobe of the right lung.

The CT chest of the patient following her surgery revealed a vascular right apical lesion ( Figure 8).

A final diagnosis of multiple hemorrhagic lesions in the brain from primary lung choriocarcinoma was eventually made. The patient was referred to the National Cancer Centre for chemotherapy. The patient was started on the EMA-CO regime (Etoposide, Methotrexate and Actinomycin by drip over 2 days, followed by Cyclophosphamide and Oncovin the following week). The patient’s B-HCG decreased sharply after the first session of chemotherapy (serum B-HCG dropped to 1,50,000 mIU/ml). The patient was given three cycles of chemotherapy and has been on regular follow up at the cancer centre.

## Discussion

Primary lung choriocarcinoma is a very rare entity, with < 50 cases reported currently ^2^. This case report discusses an even rarer phenomenon of multiple hemorrhagic metastasis in the brain from primary lung choriocarcinoma.

There are various theories behind the etiology of primary lung choriocarcinoma. The foremost being embolism of trophoblastic cells during abortion, or even normal delivery, to the lung vasculature, thereby causing the cells to proliferate therein ^3^. This may have occurred in the present case study. Other theories discuss the probable role of primordial germ cells and the genesis of metaplasia ^4^. Choriocarcinoma can have either a gestational or non-gestational origin ^5, 6^.

Sometimes large cell anaplastic carcinoma, mediastinal germ cell tumor, bronchogenic carcinoma show ectopic HCG secretion, but this elevation is mild ^7, 8^. A high B-HCG level, as in our case, suggests a trophoblastic origin ^1^.

The pathogenesis behind multiple hemorrhagic lesions in the brain is the tendency of such malignant trophoblastic cells to invade the vessels, and sometimes even leads to distal aneurysms ^9^. Radiation has a poor response to such entity ^10^. Therefore, the preferred therapy for gestational trophoblastic neoplasm is the EMA-CO regimen, similar to what was prescribed to our patient ^11^.The prognosis of the condition is poor, with previous reports of a 5 year survival of <5%. However, recent advancements in chemoradiation therapy has helped to increase the overall 5 year survival rate up to 50% ^4, 12^. A multimodal approach is also required, constituting of neo-adjuvant chemotherapy followed by excision of the lung lesion ^9^.

## Conclusions

Primary lung choriocarcinoma metastasis should be recognized as a differential diagnosis in hemorrhagic lesions of the brain, especially in patients of a child bearing age. Early diagnosis and rapid initiation of therapy is the cornerstone for a better outcome in such patients.

## Consent

Written informed consent for the publication of the clinical case study and accompanying images was taken from the patient.
