# Supplementary material for: Case Report: Multiple hemorrhagic metastases to the brain from primary lung choriocarcinoma
Source: F1000Res. 2017 May 23;6:740. [Version 1] doi: 10.12688/f1000research.11681.1 (PMC5538038; doi:10.12688/f1000research.11681.1)
Supplement: Peer review history

# Peer review: Referee response for version 1

**DOI:** 10.5256/f1000research.12619.r24086

**Published:** 2017-07-31

**Authors:** Lekhjung Thapa

**Affiliations:** 1 Neuro and Allied Clinic, Bhairahawa, Nepal

I must congratulate the author for reporting such a rare medical entity. The case has been nicely described and I am sure that this is going to be useful for other practitioners. However, I feel, few points to be considered in this case report are:

1. Sixth CN palsies may be better demonstrated on all directions of gaze. In the given picture, it looks like the patient also has BL ptosis!
2. Histopathology of lung mass if included, would be better.
3. The patient has been on follow-up at cancer center. It would be interesting to know the neurological status at follow up.
4. I think the author should discuss more with data on brain metastasis of choriocarcinoma ^1^.

I have read this submission. I believe that I have an appropriate level of expertise to confirm that it is of an acceptable scientific standard.

## References

[1] KobayashiTKidaYYoshidaJShibuyaNKageyamaN: Brain metastasis of choriocarcinoma. *Surg Neurol*.1982;17(6) :395-403 720225710.1016/s0090-3019(82)80002-5

# Peer review: Referee response for version 1

**DOI:** 10.5256/f1000research.12619.r23780

**Published:** 2017-06-26

**Authors:** Ping Wang

**Affiliations:** 1 Department of Radiotherapy, Tianjin Medical University Cancer Institute and Hospital, Tianjin, China

The paper tells us “Her immune status was normal”. I think the authors should show us the date of the immune test. Since the patient was found by multiple hemorrhagic metastases to the brain, and the primary lesion from lung, how about the tumor marker from lung? Has she had a gene test of EGFR, ROS1, ALK, T790M, which could be useful if she needed target therapy? Did she need radiation for the lung tumor and when? The author need to discuss these questions.

I have read this submission. I believe that I have an appropriate level of expertise to confirm that it is of an acceptable scientific standard.
